# Avian Influenza A(H5N1) Neuraminidase Inhibition Antibodies in Healthy Adults after Exposure to Influenza A(H1N1)pdm09

**DOI:** 10.3201/eid3001.230756

**Published:** 2024-01

**Authors:** Pavithra Daulagala, Samuel M.S. Cheng, Alex Chin, Leo L.H. Luk, Kathy Leung, Joseph T. Wu, Leo L.M. Poon, Malik Peiris, Hui-Ling Yen

**Affiliations:** School of Public Health, The University of Hong Kong, Hong Kong, China (P. Daulagala, S.M.S. Cheng, A. Chin, L.H.L. Luk, K. Leung, J.T. Wu, L.L.M. Poon, M. Peiris, H.L. Yen);; The University of Hong Kong–Shenzhen Hospital, Shenzhen, China (K. Leung);; Centre for Immunology and Infection, Hong Kong Science Park, Hong Kong (L.L.M. Poon, M. Peiris)

**Keywords:** influenza, viruses, influenza A(H5N1), influenza A(H1N1)pdm09, viruses, respiratory infections, clade 2.3.4.4.b, cross-reactive antibody response, neuraminidase inhibition antibody, pandemic risk assessment

## Abstract

We detected high titers of cross-reactive neuraminidase inhibition antibodies to influenza A(H5N1) virus clade 2.3.4.4b in 96.8% (61/63) of serum samples from healthy adults in Hong Kong in 2020. In contrast, antibodies at low titers were detected in 42% (21/50) of serum samples collected in 2009. Influenza A(H1N1)pdm09 and A(H5N1) titers were correlated.

The A/goose/Guangdong/1/1996-like (GsGD-like) highly pathogenic avian influenza A(H5N1) viruses were first identified in 1996 and have continuously evolved into antigenically distinct hemagglutinin (HA) clades that have substantially affected animal and human health. Before 2005, the GsGd-like virus mainly circulated in Asia among domestic poultry. Spillover infections from domestic poultry to wild migratory birds have enabled intercontinental spread to Europe, the Middle East, Africa, and North America, as previously observed in 2005 (clade 2.2 virus) and in 2014–2015 (clade 2.3.4.4c virus) (*1*). Since 2016, the clade 2.3.4.4b viruses have undergone a 3rd wave of intercontinental spread and have become enzootic among wild birds as of 2021 (*2*,*3*). Currently, the GsGD-like H5N1 viruses have been reported in all continents except Oceania and Antarctica. Expanded genetic diversity and geographic distribution has led to spillover events into numerous mammal species and sporadic human infections (*4*). 

Highly pathogenic avian influenza A(H5N1) virus has not yet achieved efficient transmissibility in humans, but the current epidemiology of H5N1 2.3.4.4b lineage raises concerns of possible pandemic potential. Population immunity to an emerging influenza virus is one of the key parameters considered in assessing its pandemic risk according to the Centers for Disease Control and Prevention influenza risk assessment tool (https://www.cdc.gov/flu/pandemic-resources/national-strategy/risk-assessment.htm) and the World Health Organization tool for influenza pandemic risk assessment (https://www.who.int/teams/global-influenza-programme/avian-influenza/tool-for-influenza-pandemic-risk-assessment-(tipra)). Neutralizing antibodies targeting the HA receptor-binding domain and antibodies that inhibit neuraminidase (NA) activity have been shown to correlate with protection against influenza infection (*5*,*6*). We evaluated whether healthy adults possess cross-reactive hemagglutination inhibition (HAI) and neuraminidase inhibition (NAI) antibodies to H5N1 virus through previous exposure to seasonal influenza infections.

## The Study

We collected serum samples from 63 healthy blood donors 18–73 years of age in 2020 in Hong Kong (HKU/HA HKW IRB #UW-132) to determine cross-reactive HAI antibodies and NAI antibodies to a clade 2.3.4.4b H5N1 virus (A/black-faced spoonbill/Hong Kong/AFCD-HKU-22-21429-01012/2022; Spoonbill/HK/22), which showed high homology to the HA and NA proteins of clade 2.3.4.4b candidate vaccine viruses A/chicken/Ghana/AVL-76321VIR7050-39/2021 (98.8% [HA] and 97.3% [NA]) and A/American wigeon/South Carolina/AH0195145/2021 (98.6% [HA] and 97.2% [NA]) (*7*). For comparison, we also determined the HAI and NAI antibody responses to the 2009 pandemic influenza A(H1N1)pdm09 (pH1N1) virus (A/California/04/2009; California/09) using an HAI assay and enzyme-linked lectin assay (detection limit at 1:10) (*8*,*9*).

Among healthy adults, 56/63 (88.8%) possessed HAI antibodies to pH1N1 virus with a geometric mean titer (GMT) of 21.84; none showed detectable HAI antibodies to H5N1 virus (Figure, panel A). NAI antibodies against pH1N1 were detected in 57/63 (90.5%) healthy participants (GMT 41.80), and 61/63 (96.8%) also possessed cross-reactive NAI antibodies to H5N1 (GMT 41.34) (Figure, panel B). The NAI titers against pH1N1 and H5N1 were highly correlated (Spearman ρ = 0.8349; p<0.001) (Appendix Figure 1). Furthermore, 57 (90.5%) persons had NAI antibodies to both viruses at titers >1:10, and 32 (50.8%) persons had NAI antibodies to both viruses at titers >1:40. To evaluate whether the cross-reactivity extends to N1 proteins of other avian influenza viruses, we randomly selected 32 serum samples to determine NAI titers against an avian influenza A(H6N1) virus isolated from wild bird surveillance (A/environment/Hong Kong/HKU_MPT_2006/2015; Env/HK/15). DNA barcoding suggested that the specimen originated from *Platalea minor* (black-face spoonbill). Similarly, 93.75% (30/32) persons possessed cross-reactive NAI titers against H6N1 virus (GMT 26.50), and 40.6% (13/32) possessed NAI titers >1:40 (Appendix Figure 1). However, cross-reactivity did not extend to N4 protein of an avian influenza A(H6N4) virus isolated from wild bird surveillance (A/environment/Hong Kong/HKU_MPT_2022; Env/HK/22) originated from *Anas acuta* (northern pintail) (Appendix Figure 1). Overall, we observed high correlations between NAI titers against pH1N1 and H6N1 viruses (Spearman ρ = 0.875; p<0.001) and between H5N1 and H6N1 viruses (Spearman ρ = 0.874; p<0.001).

**Figure:**
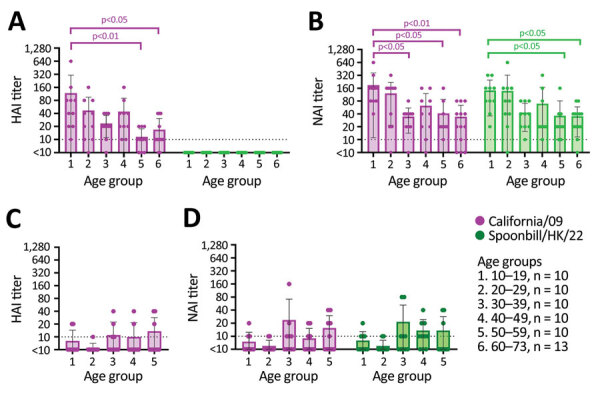
Age-stratified HAI and NAI antibody responses to influenza A(H1N1)pdm09 (California/09) and A(H5N1) (Spoonbill/HK/22) viruses in serum samples collected from healthy adults in 2020 and 2009, Hong Kong, China. A, B) Results for serum samples of 63 healthy adults collected in 2020. C, D) Results for serum samples of 50 healthy adults collected in 2009. The assay detection limit was 1:10, and samples with antibody below the detection limit were assigned an arbitrary antibody titer of 5, which is used to calculate geometric mean titer. The HAI and NAI titers across different age groups were compared using Kruskal-Wallis test and Dunn’s multiple comparison test. HAI, hemagglutination inhibition; NAI, neuraminidase inhibition.

We examined NAI titers to the homologous virus and cross-reaction to H5N1 virus in archival ferret antiserum against seasonal A(H1N1) viruses and pH1N1 (Table 1). Ferret antiserum against H1N1 viruses circulating during 1977–2007 showed no cross-reactive NAI response to H5N1 but had NAI titers at 1:320–1:1280 against the homologous viruses. Ferret antiserum against pH1N1 virus showed a homologous NAI titer of 1:2,560 and cross-reactive NAI titers to H5N1 at 1:320 to 1:640.

**Table 1:** NAI antibody titers detected in postinfection ferret antiserum against influenza A(H1N1), A(H1N1)pdm09, and A(H5N1) viruses, Hong Kong, China

Postinfection ferret antiserum†	NAI antibody titers*		
	Homologous virus	California/09 A (H1N1)pdm09	Spoonbill/HK/22 A(H5N1)
A/USSR/90/1977	320	<10	<10
A/Chile/01/1983	320	<10	<10
A/Singapore/06/1986	320	<10	<10
A/Texas/36/1991	320	<10	<10
A/Brisbane/59/2007	1,280	<10	<10
A/California/04/2009	2,560	2,560	320–640

*NAI antibody response was determined using recombinant A(H6N1) viruses carrying the N1 proteins derived from A(H1N1), A(H1N1)pdm09, or A(H5N1) viruses that were generated as described previously (*10*). Although we used recombinant H6N1 viruses in ELLA assays to avoid interference of hemagglutinin-reactive antibodies, we might not completely prevent the interference of cross-reactive antibodies that bind to the stalk of H6 protein and inhibit neuraminidase activity through stearic hindrance. NAI, neuraminidase inhibition. †One ferret antiserum against each of the influenza A(H1N1) viruses and 6 ferret antiserum against the influenza A(H1N1)pdm09 were used.

To further confirm whether exposure to pH1N1 virus contributed to the cross-reactive NAI antibodies, we used serum samples of 50 healthy blood donors (17–55 years of age) collected in July 2009, before pH1N1 had become widespread in Hong Kong (*11*). HAI antibodies to pH1N1 were detected in 22% (11/50) of the samples; GMT was low, at 7.07 (Figure, panel C). We detected NAI antibodies to pH1N1 in 40% (20/50) of healthy blood donors; GMT was low, at 8.24. NAI antibodies to H5N1 were detected in 40% (22/50) of donors; GMT was 8.35 (Figure, panel D). Most participants (16/20) with detectable NAI antibodies to pH1N1 also had NAI antibodies to H5N1. Overall, the cross-reactive NAI titers detected in 2009 (Figure, panel D) were lower than the those detected in 2020 (Figure, panel B). This result suggests that previous exposure to pH1N1 virus is the potential source of cross-reactive NAI antibodies to H5N1 virus. The NA protein of Spoonbill/HK/22 differed from the pH1N1 (California/09) NA by 53 aa and from the seasonal H1N1 NA proteins by 68–76 aa; most changes occurred in the NA head domain (Table 2; Appendix Figure 2).

**Table 2:** Comparison of the neuraminidase proteins of seasonal influenza A(H1N1) and A(H1N1)pdm09 viruses to the neuraminidase proteins of influenza A(H5N1) virus, Hong Kong, China

Viruses	No. amino acid differences compared with Spoonbill/HK/22	Amino acid homology, %
A/USSR/90/1977	70	84.08
A/Chile/01/1983	68	84.58
A/Singapore/06/1986	68	84.58
A/Texas/36/1991	69	84.33
A/Brisbane/59/2007	76	83.83
A/California/04/2009	53	88.00

## Conclusion

We detected high titers of cross-reactive NAI antibodies to clade 2.3.4.4b H5N1 virus, Spoonbill/HK/22, in samples collected from healthy adults 18–73 years of age in 2020. The N1 antibody cross-reactivity also extended to an H6N1 avian influenza virus isolated from wild bird samples in Hong Kong. Our results confirm and extend the findings from a recent study reporting cross-reactive NAI antibody responses to clade 2.3.4.4b H5N1 virus in healthy blood donors (*12*). The use of monospecific archival ferret antiserum against seasonal H1N1 and pH1N1 influenza showed that cross-reactive NAI response to H5N1 were elicited by pH1N1 but not by seasonal H1N1 viruses circulating during 1977–2007. The pH1N1 virus derived its NA protein from the avian-origin Eurasian-avian swine viruses (*13*) and appeared antigenically more closely related to the N1 of H5N1 and H6N1 avian influenza viruses but not to a N4 of H6N4 avian influenza virus. The use of serum samples collected from healthy blood donors in 2009 further confirmed that exposure to pH1N1 might have contributed to the cross-reactive NAI antibodies against H5N1.

HAI titer of >1:40 has long been established to correspond with a 50% reduction in influenza infection risk, which might be used to model the effects of cross-reactive HAI antibody titers on reducing the basic reproduction number (R_0_) of novel zoonotic viruses with pandemic potential (*14*). NAI antibodies have also been shown to protect against infection, reduce symptoms, and shorten the duration of viral shedding (*5*,*6*); however, the NAI antibody threshold that corresponds with protection has not been clearly defined.

In summary, we detected high titers of cross-reactive NAI antibodies against influenza A(H5N1) clade 2.3.4.4b virus in serum samples collected from healthy adults in 2020 but not detected in serum samples collected in 2009. Further studies are needed to confirm whether cross-reactive NAI antibodies confer protection against H5N1 infection or modulate disease severity, but our results suggest that the antibodies against H5N1 and H6N1 viruses might derive from exposure to the conserved epitopes shared between the avian-origin pH1N1 virus and avian N1 proteins. 

Appendix Additional information about avian influenza A(H5N1) neuraminidase inhibition antibodies in healthy adults after exposure to influenza A(H1N1)pdm09.
