# Supplementary material for: Avian Influenza A(H5N1) Neuraminidase Inhibition Antibodies in Healthy Adults after Exposure to Influenza A(H1N1)pdm09
Source: Emerg Infect Dis. 2024 Jan;30(1):168–71. doi: 10.3201/eid3001.230756 (PMC10756388; doi:10.3201/eid3001.230756)
Supplement: Appendix — Additional information about avian influenza A(H5N1) neuraminidase inhibition antibodies in healthy adults after exposure to influenza A(H1N1)pdm09. [file 23-0756-Techapp-s1.pdf]

Article DOI: https://doi.org/10.3201/eid3001.230756

EID cannot ensure accessibility for supplementary materials supplied by authors. Readers who have difficulty accessing supplementary content should contact the authors for assistance.

Avian Influenza A(H5N1) Neuraminidase Inhibition Antibodies in Healthy Adults after Exposure to Influenza A(H1N1)pdm09

Appendix

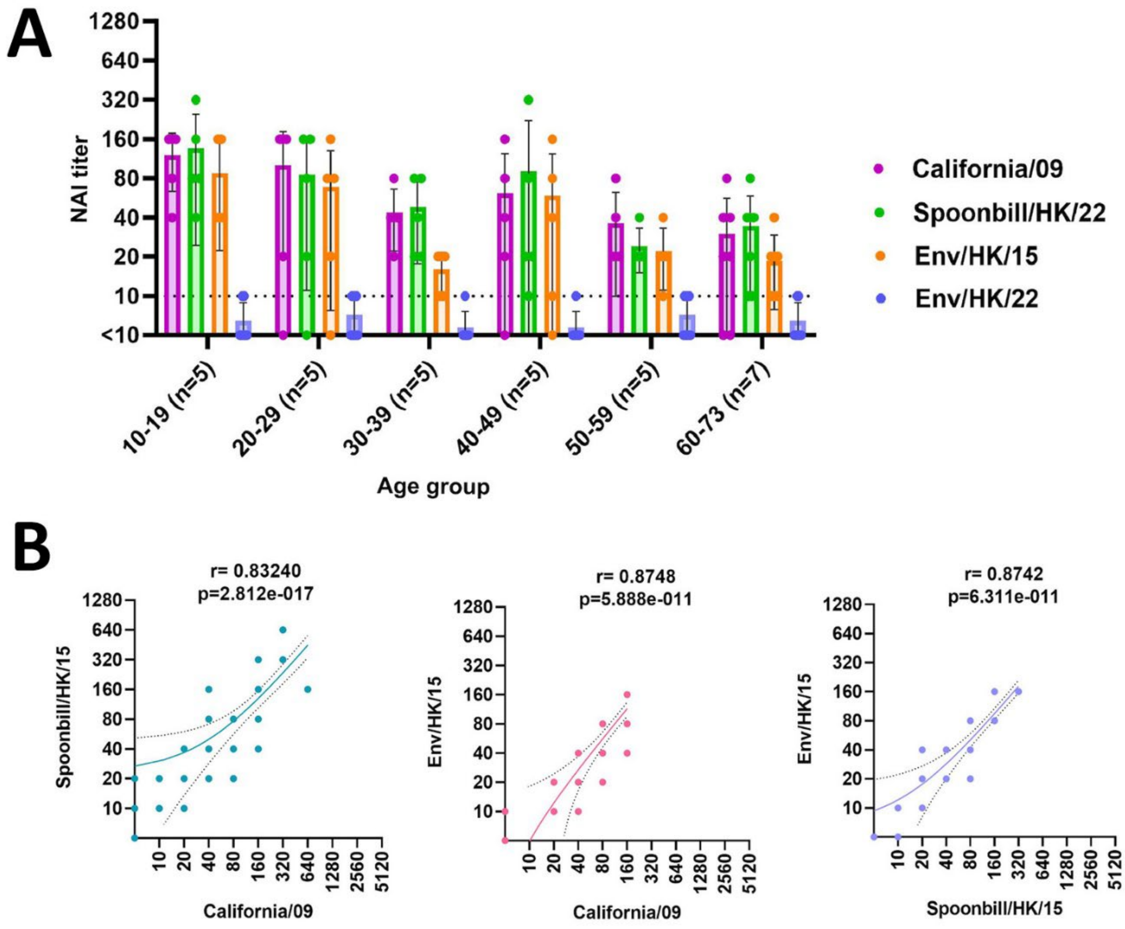

Appendix Figure 1. NAI antibody responses against an A(H6N1) avian influenza virus in healthy adults' sera collected in 2020**.** A) Age stratified NAI antibody response measured against A(H1N1)pdm09 (California/09), A(H5N1) (Spoonbill/HK/22), A(H6N1) (Env/HK/15) and A(H6N4) (Env/HK/22) from sera of

32 randomly selected healthy adults collected in 2020. The x-axis showing the age group and sample size, and the y-axis showing the HAI and NAI titers measured. The assay detection limit was 1:10 and samples with antibody below detection limit were assigned with an arbitrary antibody titer at 5, which is used to calculate GMT. The HAI and NAI titers across different age groups for each virus were compared using Kruskal Wallis test and Dunn's multiple comparison test (*p < 0.05, **p < 0.01). B) Scatter plots of the NAI antibody titers against A(H1N1)pdm09 and A(H5N1), A(H1N1)pdm09 and A(H6N1), and A(H5N1) and A(H6N1) (left to right). The Spearman's correlation coefficient and p-values are shown in each scatter plot.

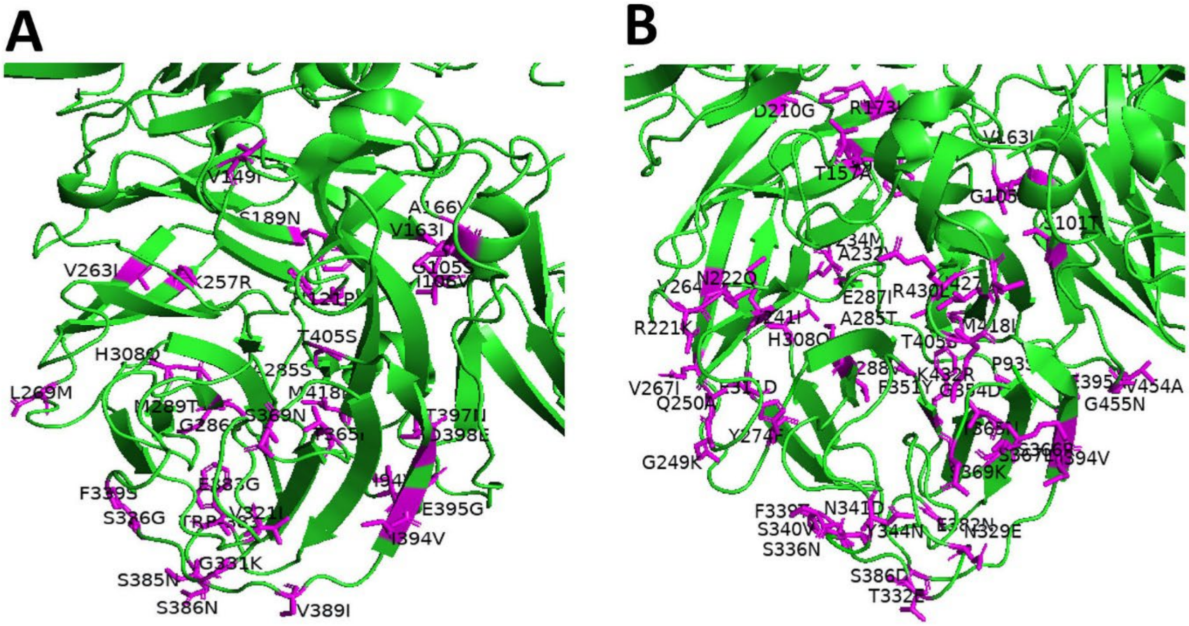

Appendix Figure 2. Amino acid substitutions on NA proteins of A(H1N1)pdm09 (California/09) and A(H1N1) (Brisbane/07) in comparison to the NA of A(H5N1) (Spoonbill/HK/22). The positions of the amino acid differences in the head region of NA are shown on the NA tetrameric structure of A/Vietnam/1203/2004 (PDB 2HU0) generated using the software PyMOL. The NA monomers are indicated in green and the amino acid substitutions are labeled in purple.
